# Assessing the influence of local environment, regional climate and tree species on radial growth in the Hexi area of arid northwest China

**DOI:** 10.3389/fpls.2022.1046462

**Published:** 2022-12-22

**Authors:** Beixi Fan, Bao Yang, Gang Li

**Affiliations:** ^1^ Key Laboratory of Desert and Desertification, Northwest Institute of Eco-Environment and Resources, Chinese Academy of Sciences, Lanzhou, China; ^2^ College of Resources and Environment, University of Chinese Academy of Sciences, Beijing, China; ^3^ School of Geography and Ocean Science, Nanjing University, Nanjing, China; ^4^ Management and Protection Centre of Gansu Qilianshan National Nature Reserve, Zhangye, China

**Keywords:** Qinghai spruce, tree-ring width, hydrological gradient, tree species, drought, Hexi area

## Abstract

Radial growth is influenced by the local environment, regional climate, and tree species. Assessing the influence of these variables on radial growth can help to reveal the relationships between tree growth and the environment. Here, we used standard dendrochronological approach to explore the response of radial growth to climate factors. We reported ring-width (TRW) residual chronologies from five sites along a longitudinal gradient in the Hexi area, arid northwestern China, based on a total of 249 Qinghai spruce (*Picea crassifolia*) ring-width records. We found that Qinghai spruce in the west of the Hexi area is more sensitive to climate change than in the east, and that drought condition in the previous growing season and the early growing season (March to June) limits spruce growth. Comparison between the regional standard chronologies of Qinghai spruce and Qilian juniper (*Juniperus przewalskii*) in the Hexi area during 1813-2001 showed that both chronologies were more consistent in the high-frequency domain than in the low-frequency domain. The findings emphasize the impacts of local environment, regional climate and tree species on radial growth, suggesting that accounting for these variables could improve large-scale and multi-species dendrochronological studies.

## Introduction

Radial growth is affected by environmental variables, including climatic factors such as temperature and precipitation (Fritts, 1976). The effects of climate change on radial growth are complex, due to spatial heterogeneity and varied responses between different tree species (Graumlich, 1993; Camarero et al., 2018; Wang and Yang, 2021). Furthermore, radial growth trends of the same tree species can be spatially variable (Danek et al., 2017; Song et al., 2020), reflecting local environmental differences such as slope, elevation, and moisture gradients (Liang et al., 2006; Kharal et al., 2014; Touchan et al., 2016; Klippel et al., 2017; Griesbauer et al., 2021; Du et al., 2022; Guerrero-Hernandez et al., 2022). However, the influences of local environment, regional climate and different tree species on radial growth are often neglected in large-scale and multi-species dendroclimatic or biomass-modeling studies. (Roy and Ravan, 1996; Cook et al., 2010).

The Hexi area is a typical arid to semi-arid region of northwest China. Forests on the mountains to the north and south of the Hexi area have the functions of water conservation, soil conservation and climate regulation (Liu et al., 2016; Deng et al., 2017). Here, Qinghai spruce (Picea crassifolia Kom) and Qilian juniper (Sabina przewalskii Kom) are endemic tree species that are both sensitive to climate change (Chen et al., 2017; Yang et al., 2021). Previous tree-ring studies of Qinghai spruce and Qilian juniper have focused on regional climate reconstruction (Yang et al., 2014; Gou et al., 2015; Yang et al., 2019; Zhang et al., 2021). Several previous studies in the Hexi area have revealed the spatial variability of radial growth along elevation gradients (Zhang and Wilmking, 2010; Gao et al., 2013; Zhang et al., 2017; Wang et al., 2019) and slopes (Liang et al., 2006; Gao, 2011). These studies suggest that climate is the main factor leading to different radial growth patterns at different sites. Most comparative studies on Qinghai spruce and Qilian juniper have focused on the differences in the response of the two species to climatic factors (Liang et al., 2006; Ran et al., 2021). However, it is not clear how climate change affects Qinghai spruce radial growth from east to west along the Hexi area. In addition, the question of consistency between the climatic signals recorded in the standard chronologies of Qilian juniper and Qinghai spruce around the Hexi area on various timescales also remains unresolved. Therefore, it is crucial to explore the effects of local environment, regional climate and tree species on the radial growth in the Hexi area.

In this study, we collected Qinghai spruce samples along an east-west transect along the Hexi area, established and compared five residual chronologies, and produced a regional Qinghai spruce residual chronology to determine the primary factors limiting radial growth in this area. Finally, we compared the regional Qinghai spruce standard chronology with a regional Qilian juniper standard chronology on multiple timescales from 1813-2001. Our study aimed to identify: (i) the main factor limiting radial growth of Qinghai spruce in the Hexi area, (ii) differences in Qinghai spruce radial growth along the east-west transect; and (iii) similarities and differences in the standard chronologies of the two dominant species (Qinghai spruce and Qilian juniper) in the Hexi area from 1813-2001.

## Materials and methods

### Study area

**Figure 1:**
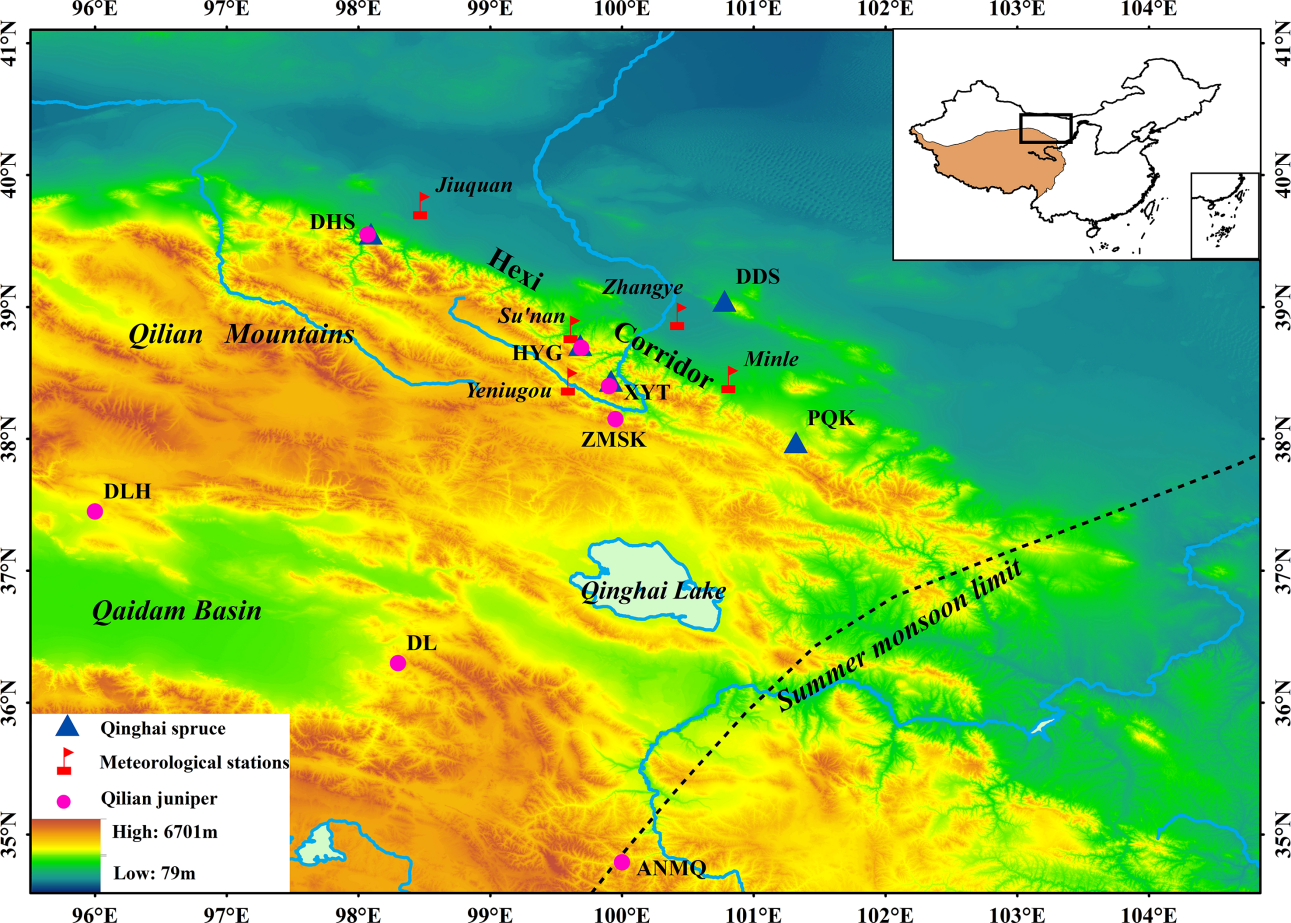
Locations of Qinghai spruce sampling sites (triangles), Qilian juniper sampling sites (circles) and meteorological stations (flags). For Qilian juniper site information, refer to Yang et al. (2010).

The study area covers a large area of Hexi area, in arid northwestern China (**Figure 1**). The Qinghai spruce sampling sites span a distance of about 1000 km from east to west. This area is characterized by a temperate continental climate. The main tree species are Qinghai spruce (*Picea crassifolia Kom*) which grow in cloudy climates on slopes between 2600 and 3100 m a.s.l (http://www.iplant.cn/frps), and Qilian juniper (*Sabina przewalskii Kom*) which grow in open stands on south-facing slopes between 3000 and 3500 m a.s.l (Chen et al., 2011; Yang et al., 2011). The forest soil is mainly mountain grey cinnamon soil (Chen et al., 2011).

### Sampling and chronology development

A total of 472 tree cores from 249 living Qinghai spruce were collected at five sampling sites (**Figure 1** and **Table S1**). Tree-ring widths (TRW) were measured using the LINTAB II platform (Germany) with an accuracy of 0.01 mm, and all the measurement series were cross-dated with TSAP software (Rinn, 2003). The quality-check of the cross-dated series was performed using the COFECHA software (Holmes, 1983).

**Figure 2:**
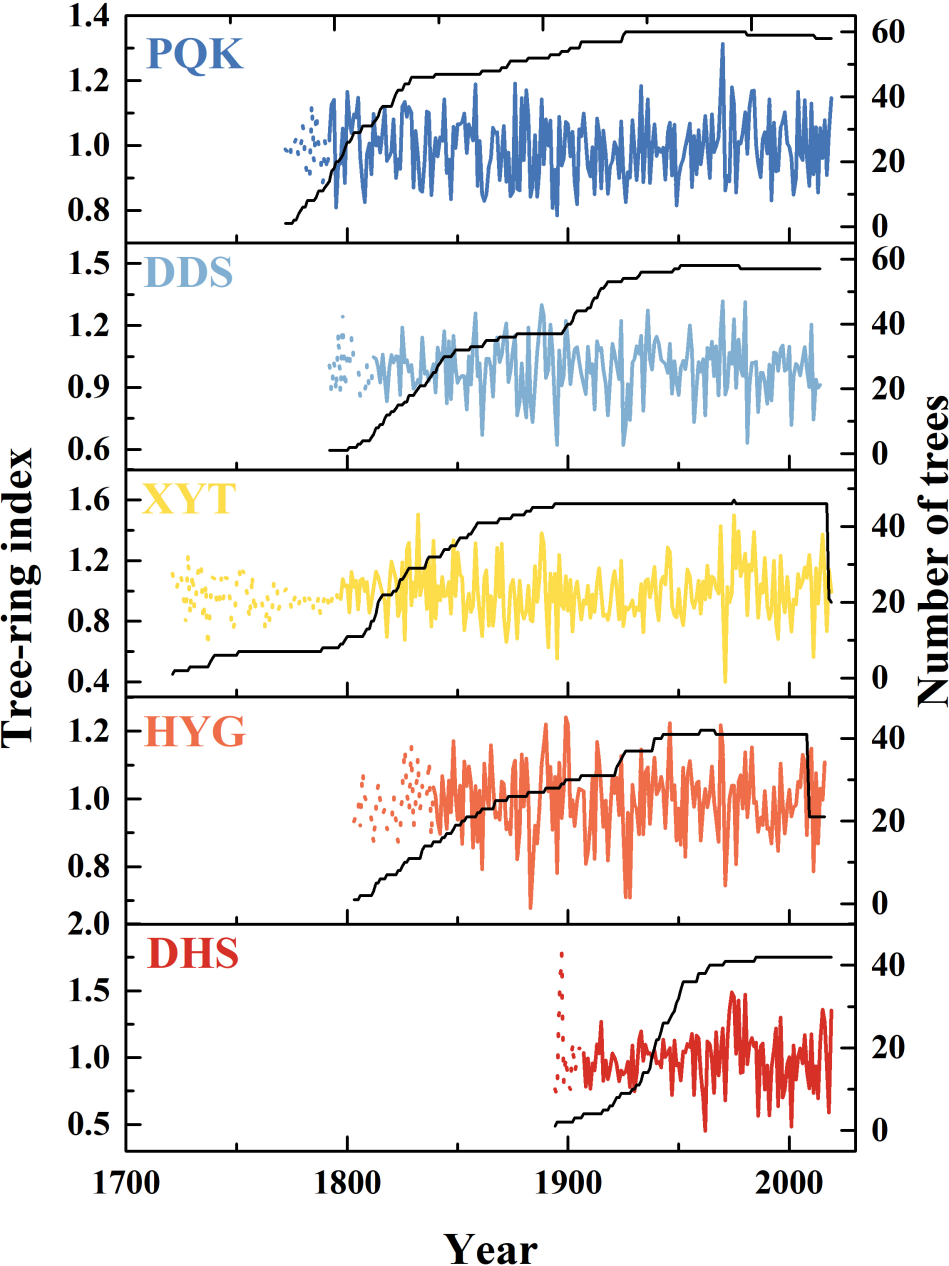
Residual chronologies and sample depths used in the study. Solid lines indicate EPS>0.85 and dotted lines indicate EPS<0.85.

The ARSTAN software was used to develop the ring-width chronologies (Cook, 1985). To remove the inherent biological age trend from the ring-width sequences, we calculated differences between the raw data and an exponential or linear growth curve. The data-adaptive power transformation was used to reduce the potential influence of outliers in the raw data (Cook and Peters, 1997). The de-trended tree-ring indices were used to establish the chronologies by using a robust bi-weighted estimate of the mean (Cook and Kairiukstis, 1990). We used the method described by Osborn et al. (1997) to stabilize the variance before calculating the final chronologies. Finally, both the standard (STD) (**Figure S1**) and residual (RES) (**Figure 2**) chronologies were produced. The STD retains low-order persistence, which is routinely used for climate reconstruction to facilitate comparison between various regions. The RES is a pre-whitened chronology in which significant persistence has been removed (Cook, 1985).

The expressed population signal (EPS) and the mean inter-series correlation (Rbar) were calculated to determine the statistically-reliable periods of the STD and RES chronologies. A 30-year moving window with 15-year overlaps was used. We selected 0.85 as the thresholds of EPS to evaluate the reliable portion of the STD and RES chronologies (Wigley et al., 1984).

### Meteorological data and statistical analysis

Monthly temperature, precipitation and relative humidity data from 1960 to 2014 were acquired from the meteorological stations (Minle, Zhangye, Yeniugou, Sunan, Jiuquan) nearest to the respective sampling sites. The self-calibrating Palmer drought severity index (scPDSI; Van der Schrier et al., 2013) is a modified variant of the Palmer drought severity index (PDSI; Palmer, 1965). We used an average series of four half-degree scPDSI grid points close to our sampling site to represent the regional moisture conditions over the period from 1960-2014.

First-order differenced climate series retain the interannual variability of climate factors. Correlation coefficients between the residual chronologies and the first-order differenced monthly values of temperature, precipitation, relative humidity and the scPDSI were calculated from 1961-2014 using the DENDROCLIM2002 software, this uses bootstrapping to assess the significance and stability of the coefficients over a specific time period (Efron, 1979; Biondi and Waikul, 2004).

Five residual chronologies with a common period (1907-2014) were selected for principal component analysis (PCA) in the SPSS (Statistical Product and Service Solutions) software (SPSS 22). Using the PCA, we explored the similarities and differences in the five residual chronologies.

To explore the high- and low- frequency variance of the Qinghai spruce and Qilian juniper chronologies, we compared their standard chronologies in the frequency domain. The Qilian juniper standard chronology was obtained from Yang et al. (2010). The regional standard chronology was produced by applying PCA to seven single-site standard chronologies from Qifeng (DHS), Sidalong (XYT), Zhamashike (ZMSK), Haiyagou (HYG), Dulan (DL), Delingha (DLH) and the Anemaqin Shan (ANMQ) (**Figure 1**). We used observations from PQK, DDS, XYT, HYG, DHS to develop a regional standard chronology of Qinghai spruce using ASTRAN (**Table S2**). Fast Fourier transform (FFT) filtering (Cooley and Tukey, 1965) was used to calculate five-year high-pass filtered data and fifty-year low-pass filtered data from the two regional standard chronologies from 1813-2001. Correlations were then calculated between the original unfiltered, first-order differenced, five-year high-pass filtered, and fifty-year low-pass filtered data. We used a Matlab program based on Monte Carlo significance tests (Macias-Fauria et al., 2012) to calculate the correlation and associated significance.

## Results

### Climate differences and chronology characteristics at the five sampling sites

**Figure 3:**
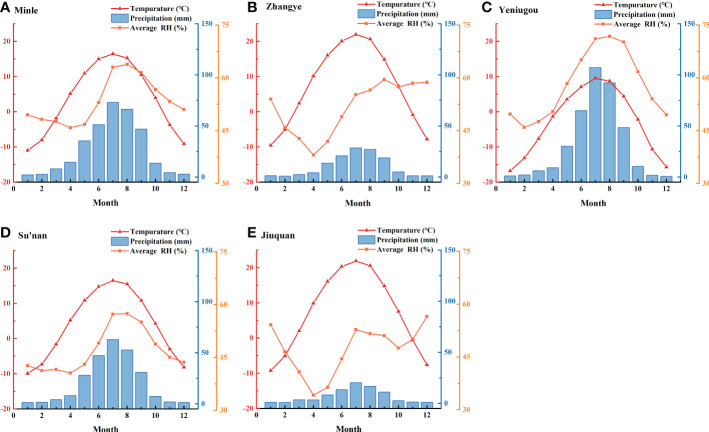
Monthly mean temperature, precipitation and relative humidity (RH) at five meteorological stations along the east-west transect in the Hexi area from 1960-2014.

The temperature, precipitation and relative humidity data during 1960-2014 from all five meteorological stations showed that temperature and precipitation were high in June, July and August, and low in December, January and February (**Figure 3**). The relative humidity was high in July, August and September and lowest in April. Under the influence of topography, the temperature gradually decreases with increasing altitude. The elevation of Yeniugou meteorological station is 3314 m, which is significantly higher than that of other sites (<2500m) (**Table S1**), and may explain why its monthly temperature was lowest and precipitation was highest. The higher relative humidity at Yeniugou may be due to the increased rainfall and decreased evaporation at high elevation. There was little change in temperature between areas of similar elevation (Minle and Sunan, Zhangye and Jiuquan), but precipitation and relative humidity gradually decreased from east to west. Overall, local climates at the five meteorological stations present similarities as well as differences, reflecting a common regional climate and the specific local environments.

Five Qinghai spruce residual chronologies were established from east to west in the Hexi area (**Figure 2**). The time spans of the PQK, DDS, XYT, HYG, DHS chronologies were 227 years (1792-2019), 202 years (1812-2014), 222 years (1797-2019), 177 years (1839-2016) and 112 years (1907-2019), respectively. The mean segment length of all samples was 188 years. All the chronologies showed low growth from 1920 to 1930 and high growth in the 1980s and after 2000.

### Principal component analysis of the five residual chronologies

**Figure 4:**
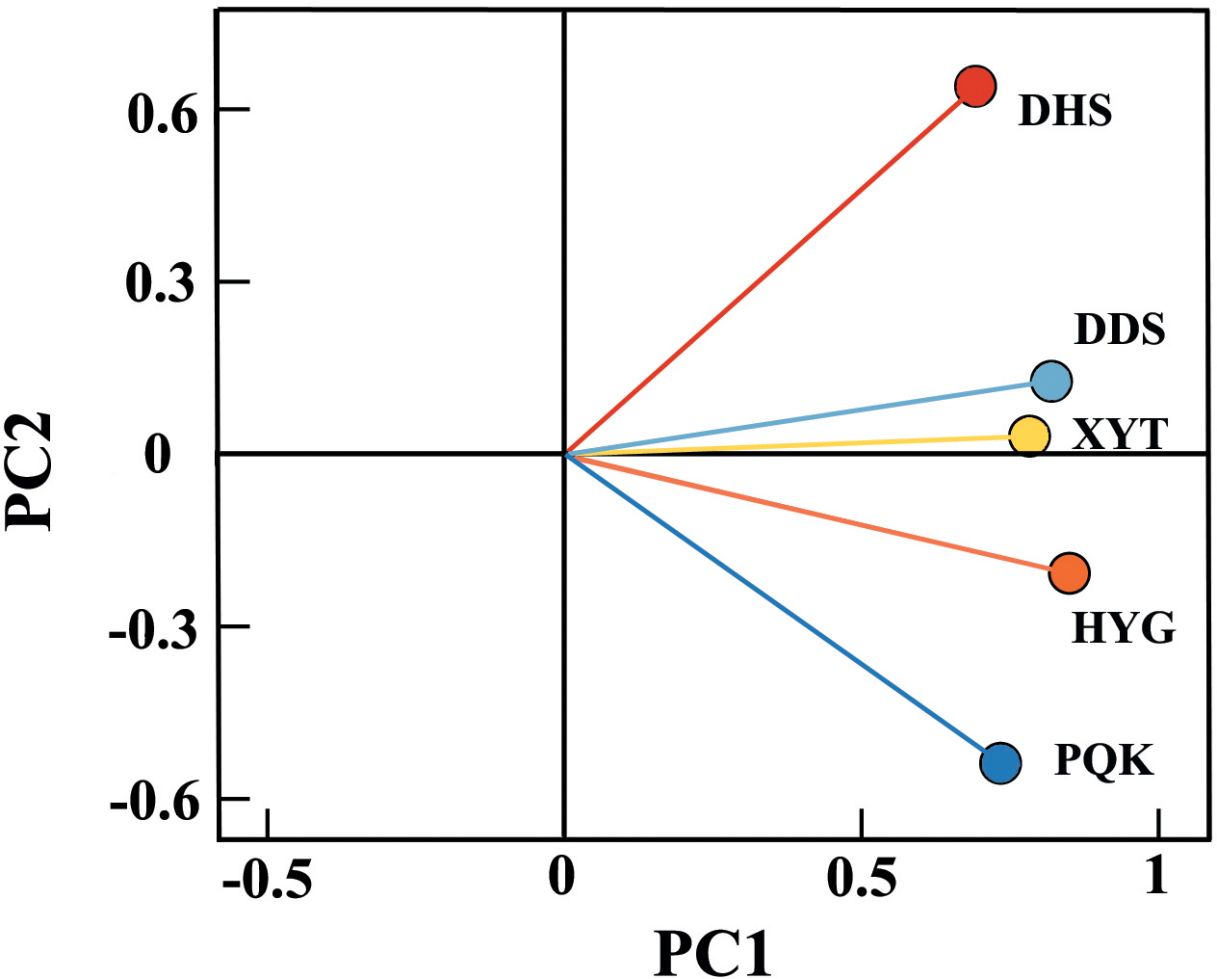
Principal component analysis of the five residual chronologies.

According to the principal component analysis of the five residual chronologies (**Figure 4**), the first principal components (PC1) of all five chronologies were between 0.5 and 1, and accounted for 60.5% of the variance, indicating that the TRW of most trees in the study area responded in the same way to environmental factors. The second principal components (PC2), accounted for 15.2%, with the easternmost PQK having a negative PC2 (-0.709) and the westernmost DHS having a positive PC2 (0.843), indicating differences between their two residual chronologies. Overall, the TRW growth status of Qinghai spruce at the five sampling sites was affected by both the regional climate and local environments.

### Statistical comparison of five local raw chronologies

**Figure 5:**
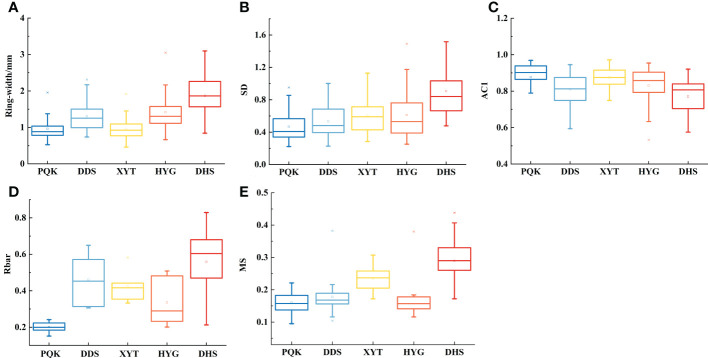
Comparison of statistics of the raw tree-ring measurements at the five sampling sites.

We compared the statistical characteristics calculated of the five raw ring-width series (**Figures 5**; **S2**). The AC1 (first-order autocorrelation) values for the five sampling sites were greater than 0.6, indicating significant low-frequency variance. The means of the SD (standard deviation), Rbar (mean inter-series correlation of all series) and MS (mean sensitivity) of the five raw TRW series were greater than 0.2, indicating considerable high-frequency variance (Fritts, 1976). Ring-width, SD, Rbar and MS were all highest, and AC1 was the lowest at the westernmost site (DHS). The opposite pattern was observed at the easternmost site (PQK). This pattern indicates greater high-frequency variance at the westernmost site (DHS) than at the easternmost site (PQK). From this we infer that tree-ring growth in drier regions is more sensitive to environmental change.

### The relationships between TRW and climate

**Figure 6:**
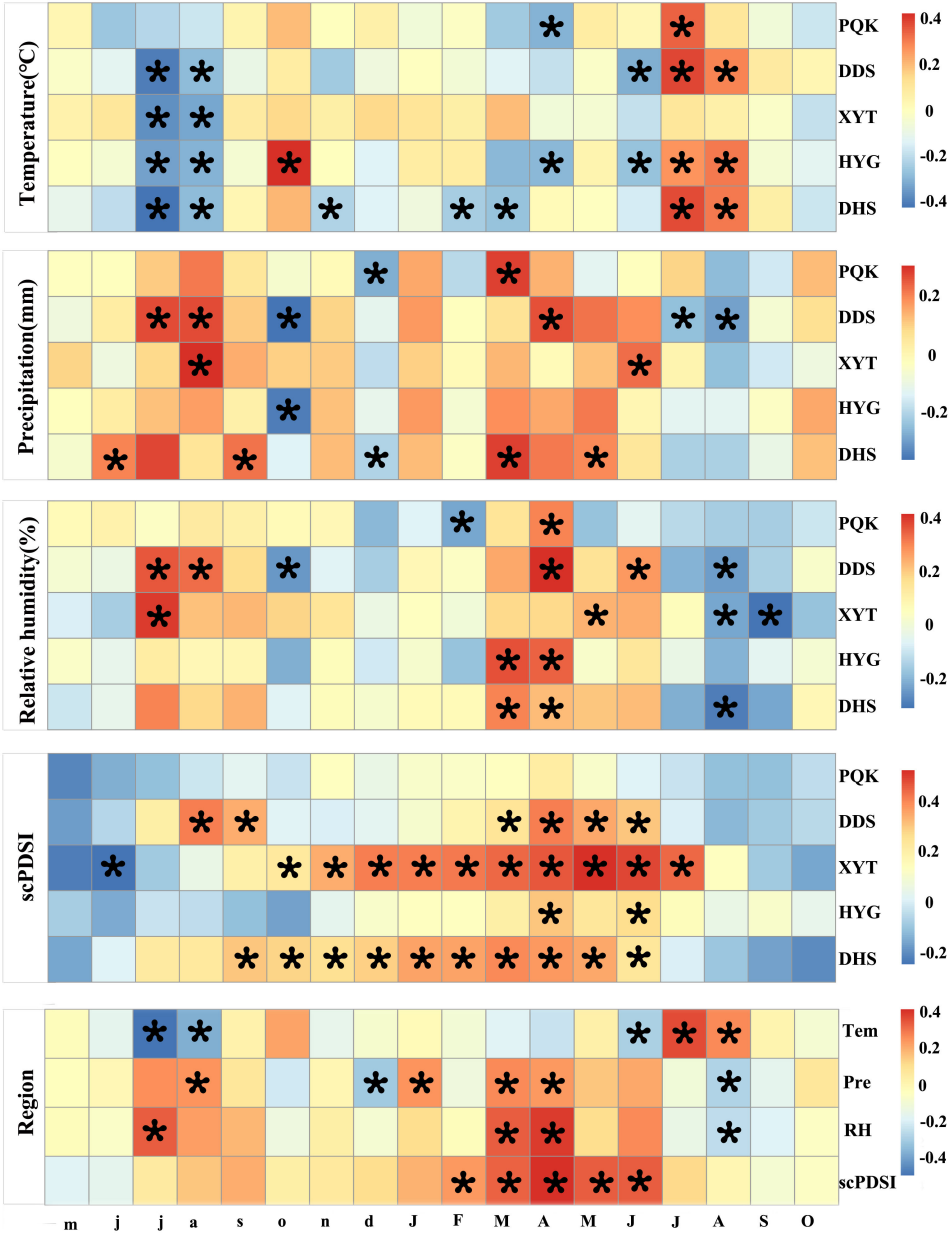
Correlations of the residual chronologies with first-order differences of mean temperature, monthly precipitation, relative humidity and scPDSI from May of the previous year to October of the current year at the five sampling sites and across the whole region (PQK, DDS, XYT, HYG, DHS) during 1961-2014. The previous year’s months are represented in lower case and those of the current year in upper case. Grid boxes with black stars indicate statistically significant results (p<0.05).

We investigated the correlations between residual chronologies and the first-order differenced climate series from May of the previous year to the current October during 1961-2014. As shown in **Figure 6**, the correlations between the five residual chronologies and temperature, precipitation, relative humidity and scPDSI were relatively consistent among the five sampling sites. The five residual chronologies showed negative correlation with the temperature in July and August of the previous year (-0.43≤r≤-0.17), while the precipitation and relative humidity were positively correlated (0.04≤r ≤ 0.39). The five residual chronologies also showed positive correlation with March to April relative humidity (0.15≤r ≤ 0.42). Correlation coefficients were much stronger with scPDSI, showing positive correlations (0.2≤r ≤ 0.53) throughout the current March to June, except for at PQK. Although PQK, located in the easternmost area, showed no significant correlation with scPDSI, it nevertheless showed positive correlation with precipitation and relative humidity in the current March to April (0.15≤r ≤ 0.31). Consequently, drought (scPDSI) from March to June is the primary limiting factor for Qinghai spruce growth in our study area. Easternmost site PQK has more precipitation and a better tree-ring growth environment, meaning there is a weaker limiting effect of drought in this area.

The correlation analysis of the five residual chronologies from 1960-2014 yielded significant positive correlations between sites (**Table S3**). The TRW measurements at PQK, DDS, XYT, HYG, DHS were selected to develop a regional TRW residual chronology that represents the growth status of Qinghai spruce across the whole region. Regional average series of temperature, precipitation (Jones and Hulme, 1996), relative humidity and scPDSI were also established. Correlation between the regional Qinghai spruce residual chronology and climate showed that regional Qinghai spruce TRW was highest when the prior summer was cool and wet. Significant positive correlation was also found between relative humidity and scPDSI in the spring and summer of the current year. These results further suggest that drought (scPDSI) in the previous growing season and early growing season (March-June) is the main limiting factor for Qinghai spruce growth.

### Comparisons with the regional Qilian juniper standard chronology

**Figure 7:**
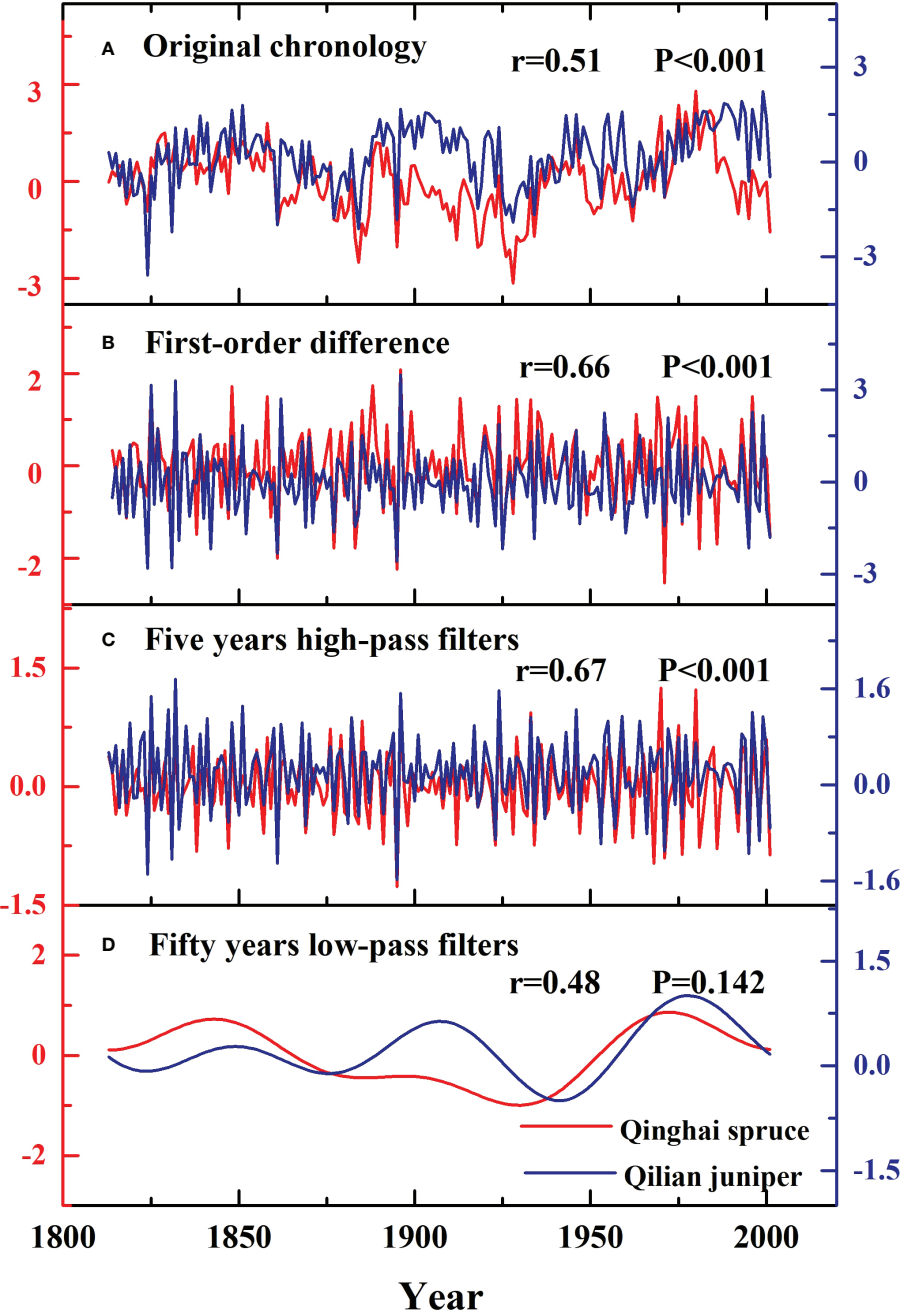
Comparison of the standard chronologies of Qinghai spruce (red lines) and Qilian juniper (blue lines) during the past 189 years.

We calculated the correlations between the standard chronologies of Qinghai spruce and Qilian juniper from 1813-2001 (**Figure 7**), finding a common response of the growth of both species to climate (r=0.51, P<0.001). Annual variability is indicated by the first-order difference of the chronology. The five-year high-pass filtered data preserve the high-frequency variability of the mean of the original series. The fifty-year low-pass filtered data preserve the relatively low-frequency variability of the mean of the original series. Overall, there was a strong correlation between the high-frequency variability of Qinghai spruce and Qilian juniper (r=0.66, r=0.67, P<0.001), while correlation between the fifty-year low-pass filtered domains was not significance (r=0.48, P=0.142).

## Discussion

### Tree-ring width response of Qinghai spruce to climate variability in the Hexi area

The five residual chronologies all showed low growth from 1920-1930 and high growth in the 1980s and after 2000 (**Figure 2**). These high and low growth trends are line with previous studies (Liu et al., 2005; Fang et al., 2009; Gao et al., 2018; Cai and Liu, 2021). Despite variations in habitat types and local environment, the PC1 of the five residual chronologies indicated a common influence of the regional climate on tree growth (**Figure 4**). This suggests that a relatively homogeneous macroclimate, independent of any differences in local ecological conditions, was the underlying cause of the high degree of covariance among the tree-ring chronologies (Peters et al., 1981; Littell et al., 2008; Liang et al., 2010).

The five chronologies showed positive correlation with precipitation and negative correlation with temperature in July and August of the previous year (**Figure 6**), suggesting that high precipitation in the previous year can promote increased nutrient transformation and storage in the tree, driving rapid growth of the cambium in the following year. At the same time, high temperatures in July and August of the previous year can cause high evaporation, resulting in poor radial growth. (Fritts, 1976). This lag between radial growth and climate suggests that the climate in the previous year may influence TRW in the next year through its effect on nutrient storage in the tree. The same relationship between climate and tree-ring growth has been reported for Qinghai spruce in the Hexi area (Chen et al., 2013) and nearby areas (Chen et al., 2011; Deng et al., 2013; Wang et al., 2016).

In our study, tree-ring growth was positively correlated with scPDSI in all months from the previous August to the current August. In particular, four consecutive months from the current March to June were significant (P<0.05) (**Figure 6**), indicating that moisture availability during these seasons is the primary limiting factor for the radial growth of Qinghai spruce. Zeng et al. (2020) used the Vaganov–Shashkin (VS) model to assess the response of Qinghai spruce stem radial growth to climate from the perspective of tree physiological processes. Their results showed that soil moisture conditions during the early growing season (May to July) significantly affected tree growth, with the early growing season accounting for > 65% of total tree-ring width index. The availability of moisture in the early growing season enhances the rate of photosynthesis, enabling the assimilation of sufficient carbohydrates to maintain a high rate of cell growth, leading to large ring widths (Zhang et al., 2016).

It is worth noting that the correlation coefficients between precipitation or relative humidity and the chronologies were relatively small. These low values may reflect differences between precipitation and relative humidity at the sampling sites and the meteorological stations. The elevations of Minle, Zhangye, Yeniugou, Sunan, and Jiuquan are 2272 m, 1484 m, 3314 m, 2311 m and 1478 m, respectively, whereas the elevation of the sampling sites ranged from 2600 m to 3100 m (**Table S1**). This result also highlights that elevation differences between meteorological stations and sampling sites can influence the analysis of relationships between radial growth and climate.

### Radial growth differences of Qinghai spruce from east to west along the Hexi area

Although some differences in the radial growth of Qinghai spruce were found from east to west, there was no consistent effect of hydrological gradients on the relationship between climate and radial growth (**Figure 6**). We found that the chronology of the westernmost region (DHS) was more sensitive to climate than that of the easternmost region (PQK), indicating that the importance of precipitation increased from east to the west along the Hexi area.

Water stress in plants results in a decrease in the total leaf surface area and leaf-level water content, increasing the sensitivity of plant growth to rainfall (Meinzer et al., 2007; Li et al., 2016; Rodgers et al., 2018). According to the principle of ecological amplitude (Fritts, 1976), the water demand of tree growth in arid areas can easily reach the limit of its physiological demand, and water is thus the main limiting factor in this area. In regions with higher precipitation, the limiting effect of precipitation on tree-ring growth is weakened and trees are less sensitive to precipitation. Therefore, the wetter PQK site in the east was less sensitive to climate than the other study sites.

### Comparison of the standard chronologies of Qinghai spruce and Qilian juniper at different timescales

We found that the standard chronologies of Qinghai spruce and Qilian juniper were consistent in the high-frequency domain (first-order difference, five-year high-pass filters) but different in the fifty-year low-pass filters (**Figure 7**). We consider that the relatively arid conditions and associated drought stress probably drive the consistent inter-annual variability of tree growth-climate relationships in the Hexi area. Division and elongation of cambium cells requires enough water to maintain normal growth (Zhang, 2018). Frequent drought conditions can lead to hydraulic failure, deficient carbon recharge, inhibited photosynthesis and thus impaired tree growth in both juniper and spruce (Mitchell et al., 2013; Choat et al., 2018).

The differences in the low-frequency climate domain between the two tree-species may be attributed to their different habitat conditions and lifespans. It is well known that the germination, growth and development of trees can vary significantly between different species due to differences in their habitats (McDowell et al., 2003; Liu et al., 2007; Brandt et al., 2014). For example, Qilian juniper can grow on sunny slopes (Yang et al., 2010). This species has the characteristics of liking sunlight, being drought-tolerant, and having a well-developed root system and strong wind resistance. In contrast, Qinghai spruce is primarily located on shady slopes with thick soil cover, low evaporation (Zheng et al., 2019), and a dense forest canopy. The different habitat conditions have led to different drought tolerance strategies in the two species. Some studies have shown that Qinghai spruce mainly resists drought by delaying dehydration, while Qilian juniper mainly resists drought by enduring dehydration (Dang et al., 2003; Ran et al., 2021). Age differences between the species can also affect the signal strength in the low-frequency domain (Konter et al., 2016). In our study, the average age of Qinghai spruce was about 180 years, and the average age of Qilian juniper was about 550 years. Differences in average age may also cause differences in the recording of climate signals. This result reminds us that when different tree species are used to reflect long-term climate change, these differences should be considered.

## Conclusion

Our tree-ring width sampling network covering the main environmental gradient of the Qinghai spruce forest ecosystems in the Hexi area and captured the spatial variability of radial growth. Here, tree growth was predominantly limited by drought (scPDSI) in the previous and early growing seasons (March-June). Increasing sensitivity of radial growth to climate along the east-west aridity gradient was also evident. Despite the physiological differences between Qinghai spruce and Qilian juniper, their standard chronologies were consistent in the high-frequency domain (first-order difference, five-year high-pass filters); however, they were different following fifty years low-pass filtering. These results may be attributed to their different habitat conditions and tree ages. Thus, the tree-ring network in the Hexi area has revealed insights into interactions between tree growth and the environment. Overall, regional climate change, the east–west aridity gradient, and tree species all played important roles in influencing radial growth. These results highlight the need to carefully consider the combined effects of these variables when performing large-scale and multi-species dendrochronological studies.

## Data availability statement

The raw data supporting the conclusions of this article will be made available by the authors, without undue reservation.

## Author contributions

BY and BF designed the study. GL provided field and data support. BF analyzed the data and wrote the first version of manuscript. BF, BY, and GL revised the manuscript and approved the submitted version. All authors contributed to the article and approved the submitted version.

## Supplementary material

Supplementary Figure 1 Standard chronologies and number of trees used in the study. Solid lines indicate EPS>0.85 and dotted lines indicate EPS<0.85.

Supplementary Figure 2 Statistical comparison of the raw tree-ring measurements at the five sampling sites (alighed by elevation).

Supplementary Table 1 Statistical information of sampling sites.

Supplementary Table 2 Correlation matrix between different pairs of five standard chronologies during the common period 1907-2014.

Supplementary Table 3 Correlation matrix between different pairs of five residual chronologies during the common period 1960-2014.
