# Supplementary figures and images for: Assessing the influence of local environment, regional climate and tree species on radial growth in the Hexi area of arid northwest China
Source: Front Plant Sci. 2022 Dec 22;13:1046462. doi: 10.3389/fpls.2022.1046462 (PMC9815462; doi:10.3389/fpls.2022.1046462)

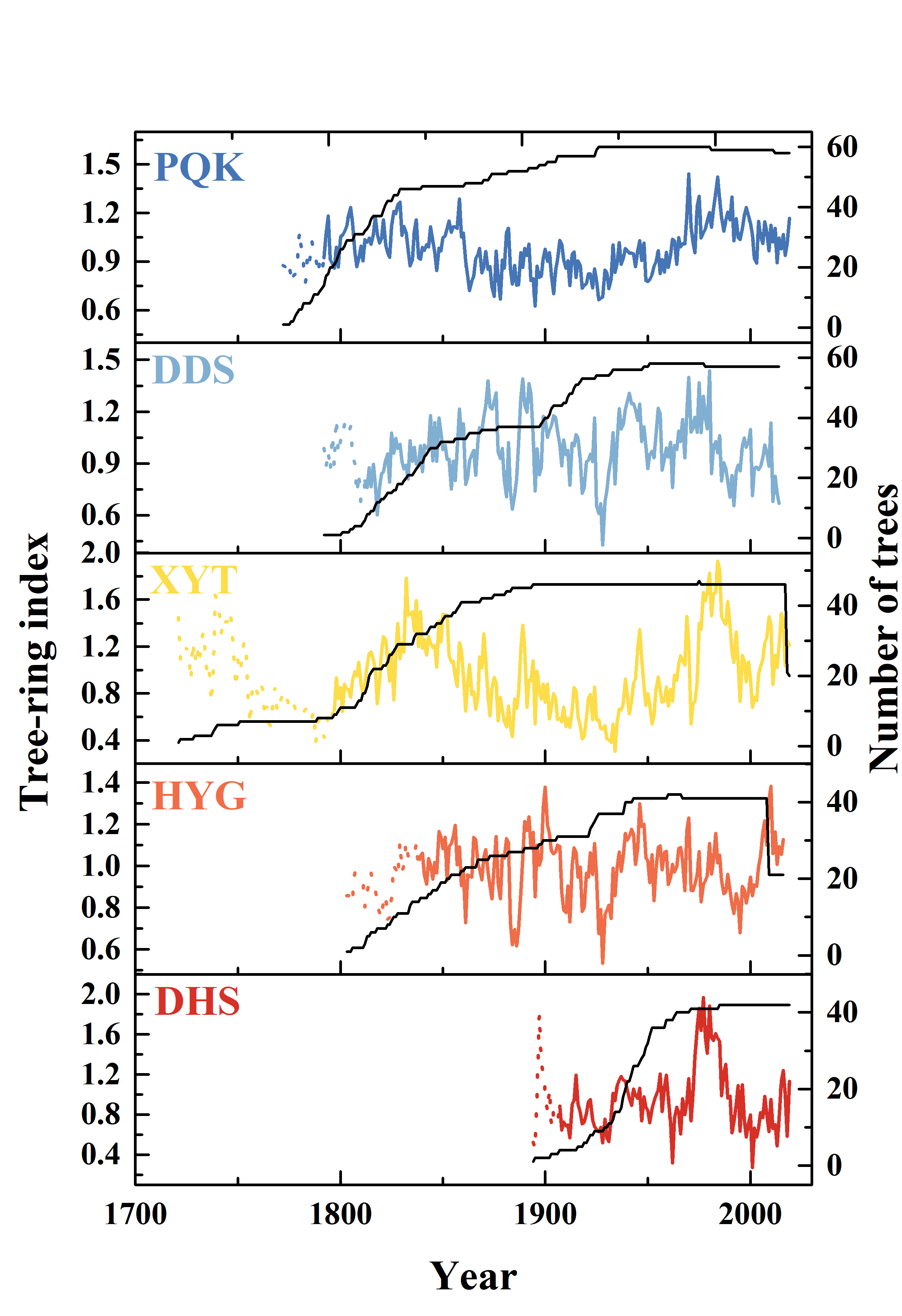

Supplement: Supplementary Figure 1 — Standard chronologies and number of trees used in the study. Solid lines indicate EPS>0.85 and dotted lines indicate EPS<0.85. [file Image_1.jpeg]

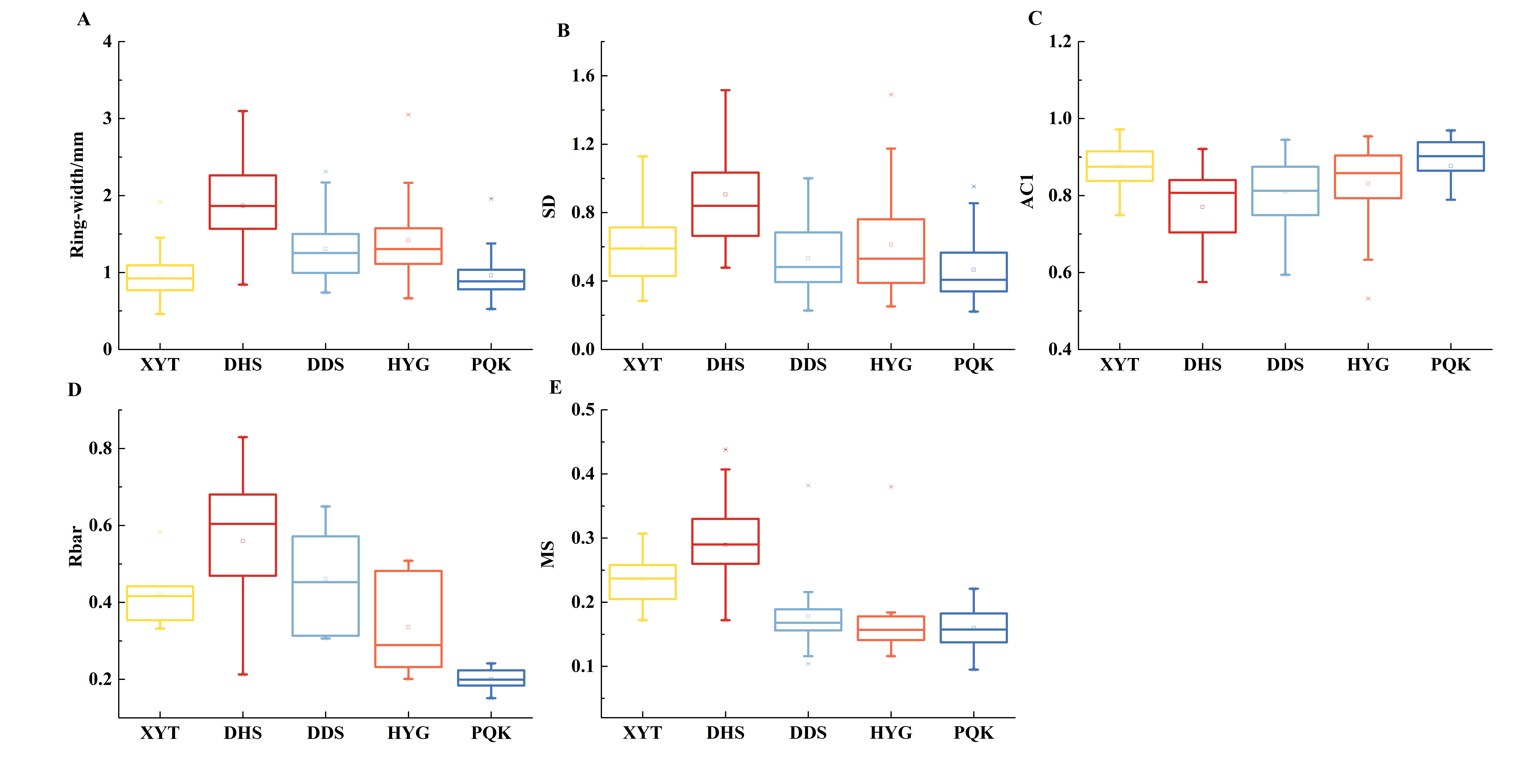

Supplement: Supplementary Figure 2 — Statistical comparison of the raw tree-ring measurements at the five sampling sites (alighed by elevation). [file Image_2.jpeg]
