# Supplementary material for: Assessing the influence of local environment, regional climate and tree species on radial growth in the Hexi area of arid northwest China
Source: Front Plant Sci. 2022 Dec 22;13:1046462. doi: 10.3389/fpls.2022.1046462 (PMC9815462; doi:10.3389/fpls.2022.1046462)
Supplement: Supplementary Table 1 — Statistical information of sampling sites. [file Table_1.docx]

**TABLE S1 |** Statistical information of sampling sites.

| Sampling site | Longitude  (E) | Latitude  (N) | Elevation  (m) | Station name | Longitude  (E) | Latitude  (N) | Elevation  (m) | Time span  (AD) | Chronology length(yr) | Mean segment length(yr) | Canopy  density |
| --- | --- | --- | --- | --- | --- | --- | --- | --- | --- | --- | --- |
| PQK | 101.319° | 37.974° | 3000-3100 | Minle | 100.82 | 38.45 | 2271.5 | 1772-2019 | 248 | 197 | 0.67 |
| DDS | 100.778° | 39.046° | 2700-3100 | Zhangye | 100.43 | 38.93 | 1483.7 | 1792-2014 | 223 | 157 | 0.63 |
| XYT | 99.915° | 38.439° | 2600-2900 | Yeniugou | 99.6 | 38.43 | 3314 | 1721-2019 | 299 | 201 | 0.61 |
| HYG | 99.682° | 38.711° | 2900-3000 | Sunan | 99.62 | 38.83 | 2311.3 | 1803-2016 | 214 | 143 | 0.53 |
| DHS | 98.093° | 39.555° | 2800-2900 | Jiuquan | 98.48 | 39.77 | 1478.2 | 1894-2019 | 126 | 81 | 0.51 |
