# Supplementary material for: Assessing the influence of local environment, regional climate and tree species on radial growth in the Hexi area of arid northwest China
Source: Front Plant Sci. 2022 Dec 22;13:1046462. doi: 10.3389/fpls.2022.1046462 (PMC9815462; doi:10.3389/fpls.2022.1046462)
Supplement: Supplementary Table 2 — Correlation matrix between different pairs of five standard chronologies during the common period 1907-2014. [file Table_2.docx]

**TABLE S2 |** Correlation matrix between different pairs of five standard chronologies during the common period

1907-2014.

|  | PQK | DDS | XYT | HYG | DHS |
| --- | --- | --- | --- | --- | --- |
| PQK | 1 |  |  |  |  |
| DDS | 0.332** | 1 |  |  |  |
| XYT | 0.762** | 0.283** | 1 |  |  |
| HYG | 0.288** | 0.566** | 0.378** | 1 |  |
| DHS | 0.448** | 0.568** | 0.588** | 0.252** | 1 |

The symbol ** denotes that the correlation is significant at the 0.01 level.
