# Supplementary material for: Assessing the influence of local environment, regional climate and tree species on radial growth in the Hexi area of arid northwest China
Source: Front Plant Sci. 2022 Dec 22;13:1046462. doi: 10.3389/fpls.2022.1046462 (PMC9815462; doi:10.3389/fpls.2022.1046462)
Supplement: Supplementary Table 3 — Correlation matrix between different pairs of five residual chronologies during the common period 1960-2014. [file Table_3.docx]

**TABLE S3 |** Correlation matrix between different pairs of five residual chronologies during the common period 1960-2014.

|  | PQK | DDS | XYT | HYG | DHS |
| --- | --- | --- | --- | --- | --- |
| PQK | 1 |  |  |  |  |
| DDS | 0.495* | 1 |  |  |  |
| XYT | 0.432* | 0.487* | 1 |  |  |
| HYG | 0.610* | 0.586* | 0.644* | 1 |  |
| DHS | 0.279* | 0.613* | 0.514* | 0.431* | 1 |

The symbol *denotes that the correlation is significant at the 0.05 level.
